# Revisiting an old idea: engineering against vector-borne diseases in the domestic environment

**DOI:** 10.1093/trstmh/try103

**Published:** 2018-09-20

**Authors:** Anne L Wilson, Mike Davies, Steve W Lindsay

**Affiliations:** 1 Department of Biosciences, Durham University, Stockton Road, Durham, UK; 2 Institute for Environmental Design and Engineering, Faculty of the Built Environment, University College London, 14 Upper Woburn Place, London, UK

**Keywords:** *Aedes*-borne diseases, built environment, housing, malaria, multi-sectoral approach, vector-borne diseases

On 21 April 1983 the Royal Society of Tropical Medicine and Hygiene held a joint meeting with the Institute of Civil Engineers at Manson House on ‘Engineering against Insect-borne Diseases in the Domestic Environment’. The summary of a talk by Chris Schofield and Graham White on ‘House design and domestic vectors of disease’ was published in a special issue of the Transactions of the Royal Society of Tropical Medicine and Hygiene.^1^ The manuscript highlights the home and peri-domestic environment as an important site of transmission for many vector-borne diseases, due to the presence of people, and in some cases animals, on which to feed, and provision of shelter from predators and extreme climate. For example, malaria mosquito vectors such as *Anopheles gambiae* readily enter houses at night to feed on humans. *Aedes aegypti*, the mosquito vector of diseases including dengue, yellow fever, Zika and chikungunya, is common in urban areas where water that collects in discarded plastic containers, car tires and water storage containers provides ideal aquatic habitats for this mosquito to lay its eggs. Cracked and uneven floors and walls can provide habitats for flea larvae, house dust mites, sandflies and triatomine bugs; the latter are vectors of leishmaniasis and Chagas disease, respectively. Flooded pit latrines, cracked septic tanks and stormwater drains provide habitats for *Culex* mosquitoes, which can transmit filariasis and contribute to nuisance biting.

Schofield and White highlighted the role of reducing hiding places for insect vectors. Examples of this included replacing mud floors with cement to reduce infestation with *Triatoma dimidiata* in Central America and exchanging palm thatch for corrugated metal roofs to control the Chagas vector, *Rhodnius prolixus*, in Venezuela. Restriction of food sources is also an option for control, including protecting stored foodstuffs against scavenging insects and the installation of proper toilets for disposal of organic waste, which attracts houseflies. Keeping domestic animals and livestock away from the home can reduce the transmission of pathogens since, for example, cats and dogs act as reservoir hosts for pathogens including *Trypanosoma cruzi* and livestock can attract sandflies such as the Indian vector *Phlebotomus argentipes* and South American vector *Lutzomyia longipalpis*. Schofield and White discussed the importance of eaves for malaria mosquito entry and the potential to reduce house entry through closing open eaves or installing a ceiling. Options for the management of vector aquatic habitats are also mentioned in the manuscript, including the provision of reliable piped water to prevent storage of water in the home, removal of water-filled receptacles and the clearance, drainage or insecticidal treatment of ponds and other water bodies.

Since the manuscript by Schofield and White was published we have seen much progress in the control of vector-borne diseases. Improvements in water and sanitation, including access to piped water, installation of latrines and removal of excreta and refuse, have led to reductions in faecal–oral infections,^2^ some of which are likely to be due to mechanical transmission of enteric pathogens by house flies. Scale-up of interventions against malaria averted 663 million malaria cases in the period between 2000 and 2015, with long-lasting insecticidal nets in particular responsible for 68% of this reduction.^3^ Despite this progress, there is still work to do to make lifesaving interventions universally available and, notably, the vector-borne disease landscape is shifting as a result of social and environmental changes. In particular, *Aedes*-borne diseases are on the rise worldwide. There were an estimated 96 million dengue cases in 2010^4^ and in recent years there have been major outbreaks of Zika, yellow fever and chikungunya.^5^ This is largely driven by urbanisation and accompanying environmental deterioration, poverty and social inequality. While there have been improvements over the past 50 years in the quality of life of millions of slum dwellers, the improvement has barely kept pace with the rate of urban growth, which is expected to double by 2050.^6^ An increase in international air travel and trade means that vector-borne diseases once confined to a particular locale now present a wider threat due to introduction of new vectors and pathogens.^7^ Spillover of pathogens from animal populations is becoming more common; Zika disease, for example, was originally a primate virus. In the past few years we have also seen stagnating progress in reducing malaria cases, due to weak vector control programmes and inadequate funding.^8^ Nowadays, vector control relies heavily on insecticidal interventions, but unfortunately these are not deployed on a sufficient scale, may be used inconsistently and the insect vectors are becoming increasingly resistant to public health insecticides.^9^ New tools and approaches are urgently needed to combat the burden of vector-borne diseases, including those outside the health sector.

The theme of controlling vector-borne diseases through the built environment is echoed in a new initiative launched last year called the BOVA (**B**uilding **O**ut **V**ector-borne diseases in sub-Saharan **A**frica) Network, which is funded by the Global Challenges Research Fund (www.bovanetwork.org). The BOVA Network aims to bring together stakeholders in the built environment and vector-borne diseases, such as architects, town planners, development practitioners, entomologists and epidemiologists, in order to stimulate research in this largely neglected discipline. The BOVA Network focuses largely on malaria and *Aedes*-borne diseases in sub-Saharan Africa, but many of the activities pertain to other vector-borne diseases. The use of multisectoral approaches to tackle vector-borne diseases is called for by the World Health Organization Global Vector Control Response^10^ and is well aligned with the Sustainable Development Goals, which cut across sectoral mandates.^11^ Major new urban policy initiatives, including the United Nation’s New Urban Agenda, are also in support of improving the urban environment to combat vector-borne diseases.^12^

The BOVA Network held their first meeting jointly with the Royal Society of Tropical Medicine and Hygiene (RSTMH) in March 2018 at University College London. The work of the BOVA Network supports the RSTMH Strategy 2017–2022, which highlights neglected tropical diseases (NTDs) and malaria as priority areas of focus and aims to strengthen partnerships across disciplines and sectors. The BOVA Network meeting showcased ongoing research in the area. For example, Charles Mbogo of the KEMRI|Wellcome Trust Research Programme explained how community-based environmental management, including education and clean-up campaigns, has helped to reduce the transmission of malaria and *Aedes*-borne diseases on the Kenyan coast.^13^ The study makes use of community mobilisation as an intervention in itself, but also emphasises that without community involvement and behaviour change, deployment of interventions will not have the desired impact or be sustainable. This has been found when failure to maintain the screening of outlet pipes on ventilated improved pit latrines led to increased fly populations.^14^ We now know that an estimated 80% or more of malaria transmission occurs when people are bitten by *Anopheles gambiae* indoors at night,^15^ so housing improvement is a major focus of the BOVA Network. For example, Lucy Tusting and Samir Bhatt from the Oxford Big Data Institute and Imperial College London are using state-of-the-art mapping techniques to capture the changing patterns of house building across sub-Saharan Africa. Ebrima Jatta and colleagues are conducting fundamental research into the house-entering behaviour of *An. gambiae* in The Gambia. These studies are providing new insights into how mosquitoes enter houses and provide important information to design houses that reduce the risk of malaria transmission. Studies of novel house improvements were presented at the meeting, including work on eave tubes against malaria.^16^ After blocking the eave spaces of houses and screening the doors and windows, small plastic tubes with insecticide-laden electrostatic netting are inserted into the house wall, below the roof. These lures, which make use of host odours to which mosquitoes are attracted, are being tested in a large trial in Cote d’Ivoire. A field study of innovative house designs in Tanzania that borrowed designs and building techniques from Southeast Asia showed that the houses reduced malaria mosquito entry while reducing indoor temperatures to encourage the use of bednets.^17^ Unfortunately, housing improvements are out of reach for many poor households in Africa and novel financing mechanisms are urgently needed. Representatives from the Centre for Affordable Housing Finance in Africa (http://housingfinanceafrica.org/) and Habitat for Humanity Terwilliger Center for Innovation in Shelter gave an overview of the housing finance market in Africa and the potential for housing microfinance.^18^

Vector-borne diseases exert a large burden of morbidity and mortality on less-developed nations and there is an urgent need for new approaches to control these diseases. Schofield and White were correct to call for an improvement in the domestic environment to fight against vector-borne diseases. With a supportive policy environment, the development of novel vector control tools and approaches and an unprecedented period of economic growth, there has never been a better time to re-examine how changes to the built environment can reduce the threat from vector-borne diseases like malaria and *Aedes*-borne diseases.
